# Development and validation of tools for predicting the risk of death and ICU admission of non-HIV-infected patients with *Pneumocystis jirovecii* pneumonia

**DOI:** 10.3389/fpubh.2022.972311

**Published:** 2022-11-08

**Authors:** Fan Jin, Hao Liang, Wen-can Chen, Jing Xie, Huan-ling Wang

**Affiliations:** ^1^Department of Infectious Diseases, Peking Union Medical College Hospital, Chinese Academy of Medical Sciences and Peking Union Medical College, Beijing, China; ^2^Clinical Pharmacology Research Center, Peking Union Medical College Hospital, Chinese Academy of Medical Sciences and Peking Union Medical College, Beijing, China; ^3^Department State Key Laboratory of Complex Severe and Rare Diseases, Peking Union Medical College Hospital, Chinese Academy of Medical Science and Peking Union Medical College, Beijing, China

**Keywords:** *Pneumocystis jirovecii* pneumonia (PCP), clinical tool, death risk, ICU admission, non-HIV

## Abstract

**Introduction:**

The mortality rate of non-HIV-infected *Pneumocystis jirovecii* pneumonia (PCP) is high. This research aimed to develop and validate two clinical tools for predicting the risk of death and intensive care unit (ICU) admission in non-HIV-infected patients with PCP to reduce mortality.

**Methods:**

A retrospective study was conducted at Peking Union Medical College Hospital between 2012 and 2021. All proven and probable non-HIV-infected patients with PCP were included. The least absolute shrinkage and selection operator method and multivariable logistic regression analysis were used to select the high-risk prognostic parameters. In the validation, the receiver operating characteristic curve and concordance index were used to quantify the discrimination performance. Calibration curves were constructed to assess the predictive consistency compared with the actual observations. A likelihood ratio test was used to compare the tool and CURB-65 score.

**Results:**

In total, 508 patients were enrolled in the study. The tool for predicting death included eight factors: age, chronic lung disease, respiratory rate, blood urea nitrogen (BUN), lactate dehydrogenase (LDH), cytomegalovirus infection, shock, and invasive mechanical ventilation. The tool for predicting ICU admission composed of the following factors: respiratory rate, dyspnea, lung moist rales, LDH, BUN, C-reactive protein/albumin ratio, and pleural effusion. In external validation, the two clinical models performed well, showing good AUCs (0.915 and 0.880) and fit calibration plots. Compared with the CURB-65 score, our tool was more informative and had a higher predictive ability (AUC: 0.880 vs. 0.557) for predicting the risk of ICU admission.

**Conclusion:**

In conclusion, we developed and validated tools to predict death and ICU admission risks of non-HIV patients with PCP. Based on the information from the tools, clinicians can tailor appropriate therapy plans and use appropriate monitoring levels for high-risk patients, eventually reducing the mortality of those with PCP.

## Introduction

*Pneumocystis jirovecii* pneumonia (PCP) is a common opportunistic infection and mostly occurs in immunocompromised individuals (1, 2). The most common cause of immunosuppression is human immunodeficiency virus (HIV) infection. However, with the popularization of standardized antiretroviral therapy and trimethoprim/sulfamethoxazole (TMP/SMX) medication for prevention and treatment purposes, the morbidity and mortality of HIV-infected patients with PCP have decreased in recent decades (3). In non-HIV-infected immunosuppressed patients, especially in patients with organ transplants (4), autoimmune diseases (5, 6), and solid-organ malignancies (7), PCP has become reproducible and increasingly prevalent (8). This may be related to the wide usage of immunosuppressants, including corticosteroids (9).

The fact that reproducible non-HIV-infected patients with PCP have presented with acute onset and more severe symptoms than HIV-infected patients with PCP is even more frustrating (2). The main pathological mechanism of PCP could be that the internal components of post-lytic *Pneumocystis jirovecii* (*P. jirovecii*), including antimicrobial lysis and immune lysis, disrupt the surfactant function of alveoli; thus, patients often present with respiratory distress (10). In non-HIV-infected patients with PCP, immune-mediated lysis of *P. jirovecii* was stronger, and the damage to surfactant function was more severe. Non-HIV-infected patients with PCP were more likely to present with many complications, including coinfection with bacteria, fungi, and viruses; pneumothorax; acute respiratory syndrome; and shock (7, 11, 12). The therapeutic plan is similar for all patients with PCP. The first-line choice is TMP/SMX monotherapy (13). Second-line therapies are considered when TMP/SMX treatment fails or results in intolerance. In addition, caspofungin could be an effective therapy for PCP (14, 15).

Both serious complications and delayed treatment can lead to high mortality. The mortality of non-HIV-infected patients with PCP could be as high as 27% (7). At present, several studies on the prognosis of patients with PCP have been published. The risk factors for death included age, pre-existing lung disease, arterial partial pressure of oxygen (PaO_2_), lactate dehydrogenase (LDH), blood urea nitrogen (BUN), albumin, pneumothorax, coinfections, delayed intubation, and delayed treatment (16, 17). However, there was neither a clinical tool available to assess the death risk of non-HIV-infected patients with PCP, partly because the number of patients with PCP was relatively small, nor was there research on the risk factors of patients with PCP who need to be transferred to the intensive care unit (ICU). The CURB-65 score is used to estimate whether outpatients with community-acquired pneumonia (CAP) need to be hospitalized or transferred to the ICU (18). The CURB-65 score comprises five factors, namely, confusion, urea, respiratory rate, blood pressure, and age over 65 years. In addition, the tool is easy for doctors to use as the five factors are frequently assessed in clinical practice. When the CURB-65 score is >3, patients should be transferred to the ICU for therapy. However, its value in non-HIV-infected patients with PCP is unclear.

Therefore, our main aim was to develop and validate clinical tools to calculate the risks of death and ICU admission in non-HIV-infected patients with PCP. The second aim of our study was to compare the performance of our tool and that of the CURB-65 score. Using to the tools, doctors can select patients with a high risk of death and tailor appropriate therapy plans for them, eventually contributing to reducing the mortality of patients with non-HIV-infected PCP.

## Patients and method

### Enrollment of participants

Patients with PCP were identified during January 2012–December 2021 at Peking Union Medical College Hospital (PUMCH), which is a 2,000-bed tertiary medical center.

In our study, only definitive cases were included. According to previous studies (19–21), PCP was proven in patients when *P. jirovecii* cysts were detected in their respiratory samples using Grocott's methenamine silver (GMS) staining or when the patients had (1) a positive PCR test for *P. jirovecii* DNA, (2) an increased level of serum β-D-glucan (BDG), (3) clinical manifestations (fever, dry cough, or dyspnea), (4) hypoxemia, and (5) radiological findings compatible with PCP. In addition, we excluded patients not receiving PCP therapy. In this study, a total of 1,083 patients were diagnosed with suspected PCP based on clinical features and radiological manifestations, but only 544 patients were included. Then, 36 patients were excluded as they were positive for HIV antibodies, were aged < 18 years, or had incomplete medical records. Finally, 508 patients were analyzed. Finally, 423 patients (January 2012–December 2020) were included to develop predictive tools (development set), and the remaining 85 patients (January 2021–December 2021) were considered the external validation set (Supplementary Figure 1).

This study was reviewed and approved by the Institutional Review Board (IRB) of PUMCH. Due to the compliance with the minimum risk exemption criteria of the IRB, the requirement to obtain informed consent from each patient was waived.

### Collection and definition of data

Baseline characteristics (age, sex, and underlying diseases), clinical data (signs, symptoms, and laboratory data upon admission), comorbidities (coinfections and shock), treatments (second-line therapy and assisted ventilation), and outcome data were extracted in detail from electronic medical records. All underlying diseases were identified according to the International Classification of Diseases, 10th Revision (ICD-10) codes. Data of chronic lung damage or kidney disease induced by autoimmune diseases were recorded. The dose of corticosteroids within 2 weeks was recorded as the prednisone equivalent. Clinical signs and symptoms included respiratory rate, heart rate, mean arterial pressure (MAP), temperature, lung moist rales, the presence of dyspnea, dry cough, and expectoration. Laboratory assessments consisted of blood assays (leukocytes, neutrophils, and lymphocytes), inflammatory indicators (C-reactive protein (CRP), CRP/albumin ratio (CAR), and erythrocyte sedimentation rate (ESR)), and liver, renal, and cardiac function. Serum BDG, PaO_2_, lymphocyte subset analysis (CD4+ T lymphocytes, CD8+ T lymphocytes, and CD4/CD8 ratio), CURB-65 scores, and the manifestations on chest computerized tomography scans were recorded.

The study had two primary endpoints: death and ICU admission. Survivors/non-survivors were defined as being alive/dead 90 days after the diagnosis of PCP. Mortality from PCP and all causes were considered death cases. The 90-day mortality and overall survival were defined as the time from diagnosis to death or the last follow-up. All data were collected by medical record review or telephone interview.

### Statistical analysis

Categorical variables were expressed as numbers (percentages) and were analyzed using the chi-square test or Fisher's exact test, as appropriate. Continuous variables were expressed as median (interquartile range) and were analyzed using the Wilcoxon signed-rank test. Missing data were imputed by the Multiple Imputation by Chained Equations (MICE) package (R package).

Based on published articles, all variables were included in the univariable analysis to first identify some potential predictive factors for the risks of death and ICU admission. The least absolute shrinkage and selection operator (LASSO) method, which can filter variables with strong collinearity, was used to select the optimal predictive factors to develop tools and compare their results with those of univariable analysis. Prognostic parameters were calculated according to the optimal cutoff values and included in the multivariable logistic regression analysis to build predicting tools. Variables with a *P*-value < 0.05 were included to develop the tools. Considering the convenient usage of the tools, continuous variables were transferred into categorical variables. Receiver operating characteristic (ROC) curve analysis and the Youden index were used to define cutoff values of the potential prognostic factors. Survival curves of every risk factor were plotted using Cox regression analysis.

In internal validation and external validation, discrimination performance and predictive accuracy were analyzed. Internal validation was conducted using 1,000 bootstrap resampling methods. The ROC curve, area under the ROC curve (AUC), and corrected Harrell concordance index (C-index) were calculated. Calibration curves were constructed to quantify the predictive consistency with the actual observation. The likelihood ratio (LR) test was used to compare the predictive performance of our tool and that of the CURB-65 score.

All statistical analyses were performed using R version 3.3.1. A *P*-value < 0.05 was considered statistically significant.

## Results

### Baseline clinical characteristics of the included patients

In total, 508 patients were included in this study, and they were divided into a development set and validation set according to the chronological order (Table 1). Some differences were found between the two sets. In particular, in the development set, 39% of patients were non-survivors, which was higher than that in the validation set (26%, *P* = 0.024).

**Table 1:** Baseline clinical characteristic of PCP cases.

**Characteristic**	**Total cases** **(*N* = 508)**	**Development set** **(*N* = 423)**	**Validation set** **(*N* = 85)**	***P* value**
Age, years	54 (42–66)	53 (40–65)	60 (52–71)	0.003
Gender, male	240 (47)	194 (46)	46 (54)	0.164
Underlying diseases				
Autoimmune disease	240 (47)	198 (47)	42 (49)	0.632
Malignancy	86 (17)	61 (14)	25 (29)	0.001
Organ transplantations	10 (2.0)	9 (2.1)	1 (1.1)	0.882
Chronic lung disease	137 (27)	96 (23)	41 (48)	< 0.001
Chronic kidney disease	162 (32)	138 (33)	24 (28)	0.428
Treatments				
TMP/SMX	500 (98)	417 (99)	83 (98)	0.878
Adjunctive steroid	379 (75)	317 (75)	62 (73)	0.699
Non-survivor	186 (37)	164 (39)	22 (26)	0.024
ICU inpatient	315 (62)	270 (64)	45 (53)	0.059

Data are presented as median (interquartile range) or number (%).

PCP, *Pneumocystis jirovecii* pneumonia; ICU, intensive care unit; TMP/SMX, trimethoprim/sulfamethoxazole; N, number of patients.

### Features of death cases and ICU admission cases in the development set

A total of 423 patients were included in the development set, among whom 164 patients were non-survivors (Table 2). The median age of the non-survivors was 60 years, which was significantly higher than that of the survivors (54 years, range 36–64 years). A greater proportion of the non-survivors had chronic lung disease, shock, and invasive mechanical ventilation (IMV); a higher respiratory rate, LDH, CRP, and CAR; and significantly lower CD8+ T lymphocyte counts (all *P* < 0.001). More non-survivors presented with a history of chronic kidney disease; showed symptoms of dyspnea, lung moist rales, and higher BUN; had lower albumin and PaO_2_ levels; had complications of CMV infection; and received second-line therapy (all *P* < 0.05).

**Table 2:** Clinical features of death PCP cases in the development set.

**Characteristic**	**Survivor** **(*N* = 259)**	**Non-survivor** **(*N* = 164)**	***P* value**
Age, years	54 (36–64)	60 (50–66)	**< 0.001**
Gender, male	117 (45)	77 (47)	0.721
BMI, kg/m^2^	23 (21–25)	23 (21–26)	0.582
Underlying diseases			
Autoimmune disease	116 (45)	82 (50)	0.295
Malignancy	32 (12)	29 (18)	0.129
Organ transplantations	5 (1.9)	4 (2.4)	0.994
Chronic lung disease	44 (17)	52 (32)	**< 0.001**
Chronic kidney disease	94 (36)	44 (27)	**0.043**
Chronic heart failure	3 (1.2)	5 (3.0)	0.306
Immunosuppression	179 (69)	107 (65)	0.407
Corticosteroids within 2 weeks, mg	482 (280–630)	434 (280–630)	0.871
Immunosuppressants	179 (69)	107 (65)	0.407
Initial clinical symptoms			
Dyspnoea	199 (77)	147 (90)	**0.001**
Dry cough	174 (67)	114 (70)	0.616
Expectoration	135 (52)	76 (46)	0.247
Initial physical sign			
Respiratory rates, bpm	22 (20–28)	27 (21–34)	**< 0.001**
Heart rates, bpm	98 (85–110)	98 (86–111)	0.526
MAP, bpm	103 (94–113)	101 (90–114)	0.166
Temperature, °C	36.3 (36.1–36.7)	36.4 (36.1–36.7)	0.804
Lung moist rales	130 (50)	110 (67)	**0.001**
Chest radiography findings			
Bilateral GGOs	201 (78)	124 (76)	0.635
Pleural effusion	93 (36)	47 (29)	0.123
Pneumothorax	19 (7.3)	20 (12)	0.092
Laboratory findings			
Leukocytes (×10^9^/L)	8.0 (5.6–11.3)	7.7 (5.4–11.8)	0.971
Neutrophils (×10^9^/L)	6.9 (4.7–9.7)	7.3 (5.1–10.3)	0.537
Lymphocyte (×10^9^/L)	0.57 (0.37–1.02)	0.44 (0.27–0.82)	0.1
Hemoglobin, g/l	111 (96–128)	109 (90–125)	0.258
Albumin, g/L	29 (26–32)	27 (24–30)	**0.007**
TBIL, mg/dl	8 (6–12)	11 (8–16)	0.287
LDH, U/L	515 (366–637)	604 (489–792)	**< 0.001**
BUN, mmol/L	6.6 (5.1–9.0)	8 (6–13)	**0.001**
Cr, μmol/L	71 (56–106)	79 (56–112)	0.911
PaO_2_, mmHg	67 (55–85)	60 (52–76)	**0.013**
CRP, mg/dL	42 (17–107)	82 (33–157)	**< 0.001**
ESR, mm/h	50 (28–81)	50 (34–82)	0.496
CAR	1.49 (0.57–3.73)	3.15 (1.16–6.51)	**< 0.001**
NT–proBNP, pg/mL	414 (132–1,219)	724 (257–2,089)	0.271
BDG, pg/mL	521 (165–1,212)	597 (232–1,361)	0.12
CD4 T lymphocyte, cells/mm^3^	131 (62–274)	103 (38–175)	0.18
CD8 T lymphocyte, cells/mm^3^	179 (95–400)	126 (70–225)	**< 0.001**
CD4/CD8 ratio	0.78 (0.42–1.48)	0.70 (0.36–1.14)	0.487
Complications and treatments			
CMV infection	135 (52)	106 (65)	**0.011**
Bacterial infection	105 (41)	74 (45)	0.353
Fungi infection	47 (18)	42 (26)	0.067
Shock	39 (15)	91 (55)	**< 0.001**
Adjunctive steroid therapy	238 (92)	149 (90)	0.709
Second-line therapy	53 (20)	50 (30)	**0.019**
IMV	110 (42)	147 (90)	**< 0.001**
ECMO	3 (1.2)	15 (9.1)	0.923

Data are presented as median (interquartile range) or number (%).

BMI, body mass index; bpm, beat per minute; MAP, mean arterial pressure; GGOs, ground glass opacities; TBIL, total bilirubin; LDH, lactate dehydrogenase; BUN, blood urea nitrogen; Cr, creatinine; PaO_2_, room air arterial partial pressure of oxygen; CRP, C-reactive protein; ESR, erythrocyte sedimentation rate; CAR, C-reactive protein/albumin ratio; NT-proBNP, N-terminal pro-brain natriuretic peptide; BDG, β-D-glucan; CMV, cytomegalovirus; IMV, invasive mechanical ventilation; ECMO, extracorporeal membrane oxygenation; N, number of patients. The bold values indicates the value of *p* < 0.05 and the difference which are considered as statistically significant.

A total of 270 patients were transferred to the ICU. The difference in age between the ICU inpatients and non-ICU inpatients was not statistically significant (Table 3). More ICU inpatients had dyspnea, lung moist rales, and pleural effusion; significantly higher respiratory rate, heart rate, LDH, BUN, CRP, and CAR; and significantly lower lymphocyte, albumin, and CD8+ T lymphocyte counts (all *P* < 0.05). In addition, the CURB-65 scores of every patient were calculated. The results showed that the difference between the ICU inpatients and non-ICU inpatients was not statistically significant.

**Table 3:** Clinical features of ICU admission cases in the development set.

**Features**	**Non-ICU inpatient** **(*N* = 153)**	**ICU inpatient** **(*N* = 270)**	***P* value**
Age, years	55 (38–64)	58 (42–65)	0.283
Gender, male	74 (48)	120 (44)	0.437
BMI, kg/m^2^	22 (20–24)	23 (21–26)	0.093
Underlying diseases			
Autoimmune disease	73 (48)	125 (46)	0.779
Malignancy	24 (16)	37 (14)	0.577
Organ transplantations	3 (2.0)	6 (2.2)	0.858
Chronic lung disease	35 (23)	61 (23)	0.947
Chronic kidney disease	41 (27)	97 (36)	0.054
Chronic heart failure	3 (2.0)	5 (1.9)	0.937
Immunosuppression	103 (67)	183 (68)	0.923
Corticosteroids within 2 weeks, mg	420 (224–630)	490 (320–630)	0.765
Immunosuppressants	103 (67)	183 (68)	0.923
Initial clinical symptoms			
Dyspnoea	103 (67)	243 (90)	**< 0.001**
Dry cough	100 (65)	188 (70)	0.365
Expectoration	83 (54)	128 (47)	0.176
Initial physical sign			
Respiratory rates, bpm	20 (20–23)	26 (21–33)	**< 0.001**
Heart rates, bpm	94 (83–102)	100 (88–112)	**< 0.001**
MAP, bpm	103 (95–111)	103 (91–115)	0.919
Temperature, °C	36.4 (36.1–36.7)	36.4 (36.1–36.7)	0.706
Lung moist rales	70 (46)	170 (63)	**0.001**
Chest radiography findings			
Bilateral GGOs	115 (75)	210 (78)	0.54
Pleural effusion	38 (25)	102 (38)	**0.007**
Pneumothorax	13 (8.5)	26 (9.6)	0.699
Laboratory findings			
Leukocytes (×10^9^/L)	8.1 (5.5–11.2)	7.8 (5.5-11.6)	0.676
Neutrophils (×10^9^/L)	6.8 (4.5–9.6)	7.2 (5.1–10.1)	0.158
Lymphocyte (×10^9^/L)	0.64 (0.36–1.04)	0.48 (0.30–0.84)	**0.033**
Hemoglobin, g/L	114 (95–132)	109 (92–124)	0.071
Albumin, g/L	31 (26–34)	27 (24–30)	**< 0.001**
TBIL, mg/dl	8 (6–12)	10 (7–15)	0.207
LDH, U/L	460 (335–593)	592 (474–792)	**< 0.001**
BUN, mmol/L	6.5 (5.2–8.7)	8 (5–12)	**0.006**
Cr, μmol/L	70 (56–104)	75 (56–113)	0.189
PaO_2_, mmHg	67 (56–82)	62 (52–80)	0.589
CRP, mg/dL	35 (12–71)	79 (31–156)	**< 0.001**
ESR, mm/h	49 (29–77)	52 (31–82)	0.17
CAR	1.16 (0.35–2.46)	2.9 (1.1–6.0)	**< 0.001**
NT–proBNP, pg/mL	401 (121–1,239)	604 (197–1,656)	0.463
BDG, pg/mL	483 (143–1,128)	649 (231–1,452)	0.064
CD4 T lymphocyte, cells/mm^3^	141 (62–314)	111 (52–184)	0.135
CD8 T lymphocyte, cells/mm^3^	208 (115–407)	136 (75–252)	**< 0.001**
CD4/CD8 ratio	0.78 (0.36–1.44)	0.72 (0.41–1.33)	0.197
CURB-65 scores			
0	26 (17)	37 (14)	0.361
1	50 (33)	72 (27)	0.19
2	45 (29)	85 (31)	0.658
3	25 (16)	55 (20)	0.309
4	7 (4.6)	19 (7.0)	0.311
5	0	2 (0.7)	0.537

Data are presented as median (interquartile range) or number (%).

ICU, intensive care unit; BMI, body mass index; bpm, beat per minute; MAP, mean arterial pressure; GGOs, ground glass opacities; TBIL, total bilirubin; LDH, lactate dehydrogenase; BUN, blood urea nitrogen; Cr, creatinine; PaO_2_, room air arterial partial pressure of oxygen; CRP, C-reactive protein; ESR, erythrocyte sedimentation rate; CAR, C-reactive protein/albumin ratio; BNP, N-terminal pro-brain natriuretic peptide; BDG, β-D-glucan; CURB-65 scores, total scores of the confusion, uremia, elevated respiratory rate, hypotension, and age≥65 years; N, number of patients. The bold values indicates the value of *p* < 0.05 and the difference which are considered as statistically significant.

### Independent risk factors for death in patients with PCP

**Figure 1:**
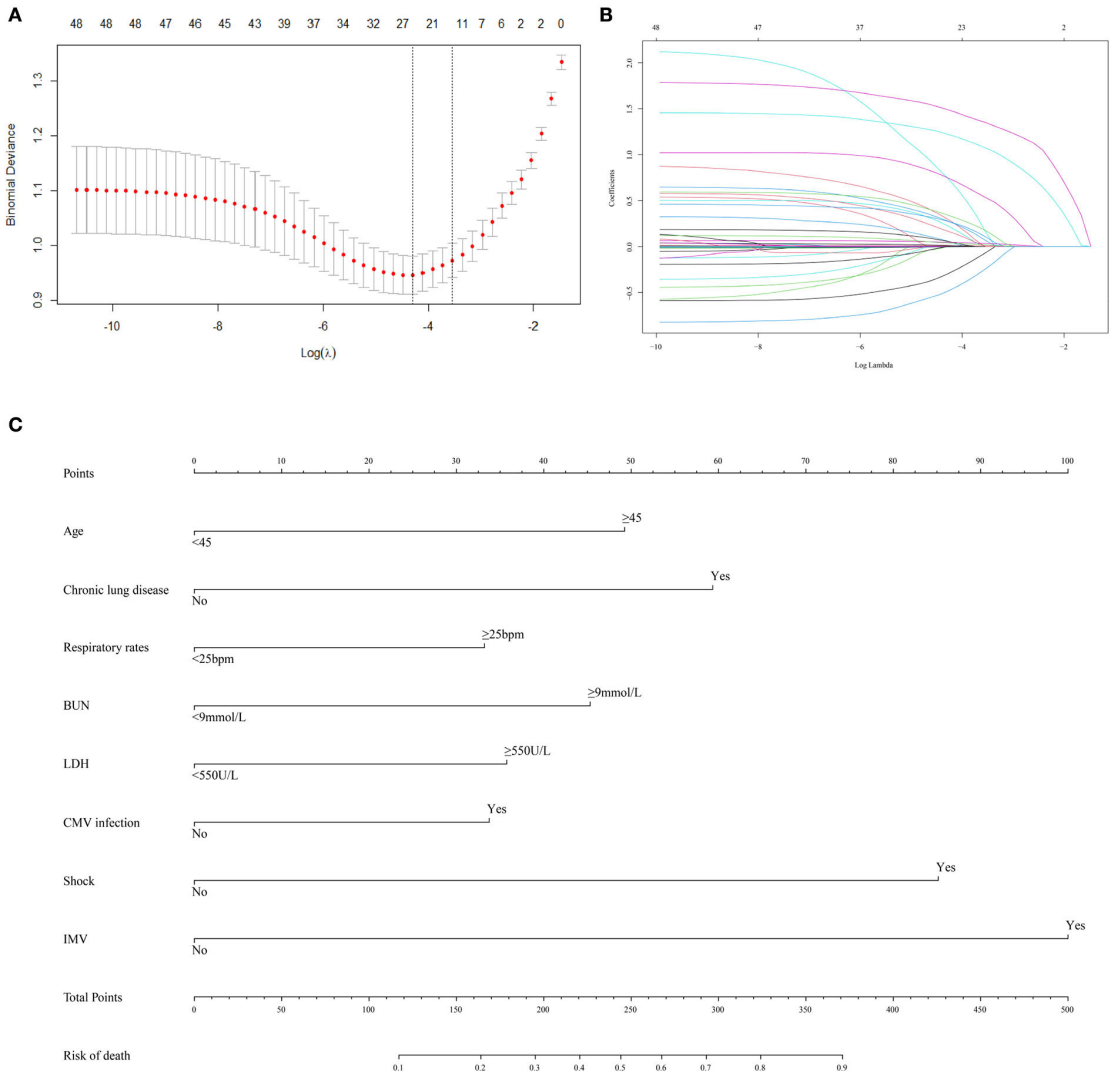
Predictor selection and prognostic nomogram development for predicting death risk of patients with PCP. **(A)** Identification of optimal parameters (lambda) in the LASSO model using minimum criteria and 5-fold cross-validation. Dotted vertical lines are drawn at the selected values using the minimum criteria and the 1 standard error of the minimum criteria (1-SE criteria); **(B)** LASSO coefficient profiles of 48 features; **(C)** predictor scores were found on the uppermost point scale that matched with the value of each variable. The sum of these scores is located on the total points axis, and a line is drawn straight down to get the percent probability of death. LASSO, least absolute shrinkage and selection operator; bpm, beat per minute; BUN, blood urea nitrogen; LDH, lactate dehydrogenase; CMV, cytomegalovirus; IMV, invasive mechanical ventilation.

A total of 48 variables, including demographic, clinical, and treatment features, were included in the LASSO regression (Figures 1A,B). When the optimal lambda was 0.035, the number of prognostic factors was reduced to 14. These factors also showed significant differences between the non-survivors and survivors in the univariable analysis (Table 2). Considering the ease of use and clinical advice, the continuous variables, including age, respiratory rate, BUN, and LDH, were converted into categorical variables. The cutoff values of age, respiratory rate, BUN, and LDH were 45 years, 25 beats per minute (bpm), 9 mmol/L, and 550 U/L, respectively (Table 4). The AUCs, specificity, and sensitivity of all factors were given.

**Table 4:** ROC curve analysis and multivariable logistic regression analysis in selected factors of death of patients with PCP identified by LASSO regression.

**Variables**	**ROC curve analysis**				**Multivariable logistic**		
					**regression analysis**		
	**AUC**	**Cutoff**	**Specificity**	**Sensitivity**	**β**	**Odds ratio (95%CI)**	***P* value**
Age (years), ≥45 vs. < 45	0.614	46.5	0.41	0.79	0.826	2.284 (1.197–4.433)	**0.013**
Chronic lung disease, yes vs. no	0.574	–	0.83	0.32	1.114	3.047 (1.603–5.928)	**< 0.001**
Dyspnoea, yes vs. no	0.564	–	0.23	0.90	0.498	1.645 (0.738–3.772)	0.230
Lung moist rales, yes vs. no	0.584	–	0.50	0.67	−0.134	0.874 (0.491–1.543)	0.645
Respiratory rates (bpm), ≥25 vs. < 25	0.650	24.5	0.66	0.60	0.580	1.785 (1.038–3.082)	**0.036**
BUN (mmol/L), ≥9 vs. < 9	0.604	9.2	0.76	0.46	0.832	2.299 (1.341–3.982)	**0.003**
CAR (mg/g), ≥2.5 vs. < 2.5	0.632	2.58	0.65	0.57	0.253	1.288 (0.751–2.201)	0.356
LDH (U/L), ≥550 vs. < 550	0.655	533.5	0.53	0.71	0.547	1.728 (1.008–2.984)	**0.048**
PaO2 (mmHg), ≥60 vs. < 60	0.582	62.25	0.60	0.58	−0.516	0.597 (0.351–1.008)	0.055
CD8 T lymphocyte (cells/mm^3^), ≥100 vs. < 100	0.622	138.5	0.63	0.55	0.278	1.321 (0.763–2.310)	0.324
CMV infection, yes vs. no	0.550	–	0.48	0.65	0.543	1.722 (1.022–2.926)	**0.042**
Shock, yes vs. no	0.700	–	0.85	0.55	1.506	4.507 (2.572–8.081)	**< 0.001**
Second–line therapy, yes vs. no	0.563	–	0.80	0.30	0.566	1.762 (0.992–3.157)	0.055
IMV, yes vs. no	0.736	–	0.58	0.90	1.748	5.746 (2.981–11.567)	**< 0.001**

ROC, receiver operating characteristic; PCP, *Pneumocystis jirovecii* pneumonia; LASSO, least absolute shrinkage and selection operator; AUC, area under curve; bpm, beat per minute; CI, confidence interval; BUN, blood urea nitrogen; CAR, C-reactive protein/albumin ratio; LDH, lactate dehydrogenase; PaO_2_, room air arterial partial pressure of oxygen; CMV, cytomegalovirus; IMV, invasive mechanical ventilation. The bold values indicates the value of *p* < 0.05 and the difference which are considered as statistically significant.

Furthermore, all 14 factors were subsequently included in the multivariate logistic regression analysis. The results revealed that age (OR = 2.284, 95% CI: 1.197–4.433, *P* = 0.013), chronic lung disease (OR = 3.047, 95% CI: 1.603–5.928, *P* < 0.001), respiratory rate (OR = 1.785, 95% CI: 1.038–3.082, *P* = 0.036), BUN (OR = 2.299, 95% CI: 1.341–3.982, *P* = 0.003), LDH (OR = 1.728, 95% CI: 1.008–2.984, *P* = 0.048), CMV infection (OR = 1.722, 95% CI: 1.022–2.926, *P* = 0.042), shock (OR = 4.507, 95% CI: 2.572–8.081, *P* < 0.001), and IMV (OR = 5.746, 95% CI: 2.981–11.567, *P* < 0.001) were independent prognostic factors affecting the outcome of patients with PCP. In addition, the Cox regression analysis of these eight independent prognostic factors demonstrated that these factors could significantly affect the survival of patients with PCP (Supplementary Figures 2A–H).

### Construction and validation of a clinical tool for predicting death risk

The eight selected independent risk factors were used to develop a clinical tool to predict death. The tool is displayed as a nomogram in Figure 1C. According to the prognosis nomogram, the death risk of patients with PCP would be >50% when they had a history of chronic lung disease, experienced shock, and received IMV.

**Figure 2:**
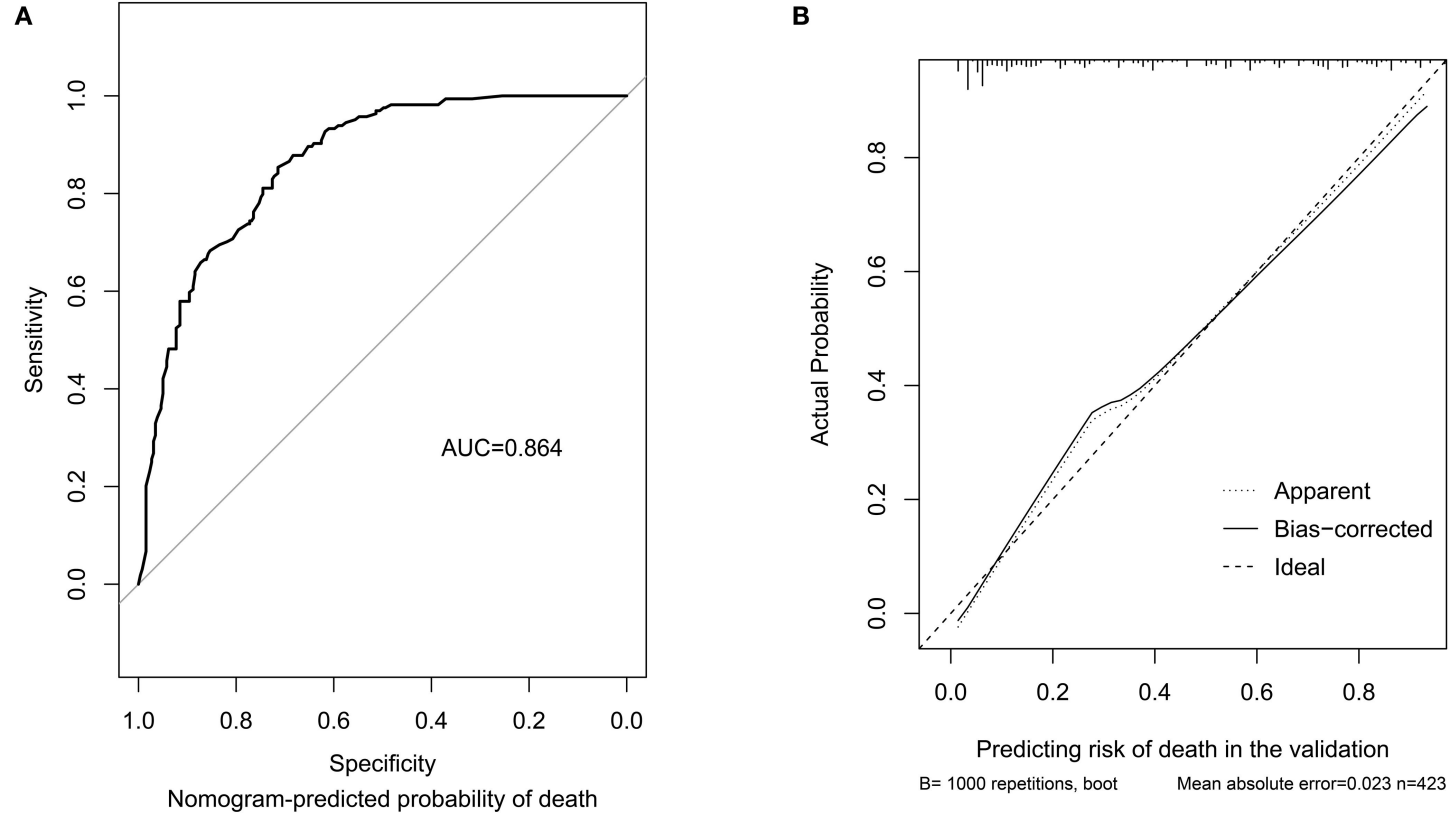
Discrimination performance and predictive consistency of the tool for predicting death risk in internal validation. **(A)** ROC curve and AUC of the tool for predicting probability of death; **(B)** calibration curves of the tool predicting probability of death. The dotted line represents a perfect prediction by an ideal model, and the solid line represents the performance of the nomogram in the validation. A closer fit of the lines represents a better prediction. ROC, receiver operating characteristic; AUC, area under curve; ICU, intensive care unit.

In the internal validation, the C-index was 0.85 for the 1,000 samples from bootstrap resampling, and the AUC value of this tool was 0.864 (95% CI: 0.830–0.898), as shown in Figure 2A. The ideal C-index and AUC value showed good discrimination. The calibration curve is illustrated in Figure 2B, which demonstrates that the predictive consistency of death risk of the nomogram was consistent with the actual death risk of patients with PCP.

**Figure 3:**
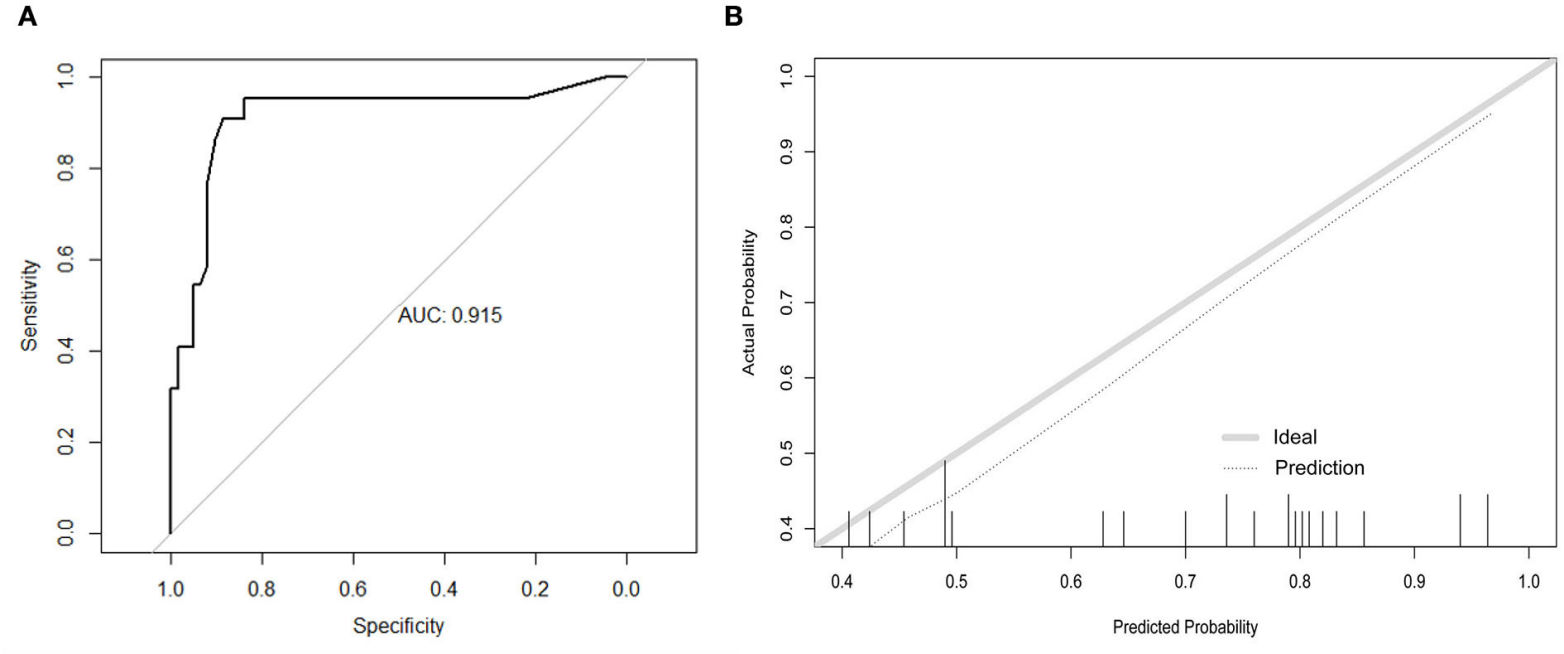
Discrimination performance and predictive consistency of tools for predicting death in the external validation. **(A)** ROC curve and AUC of the tool for predicting probability of death; **(B)** calibration curves of the tool predicting probability of death. The dotted line represents a perfect prediction by an ideal model and the solid line represents the performance of the nomogram in the validation. A closer fit of the lines represents a better prediction. ROC, receiver operating characteristic; AUC, area under curve; ICU, intensive care unit.

In the external validation (Figure 3A), the AUC value of the nomogram was 0.915 (95% CI: 0.831–0.997), which was higher than that in the internal validation. The calibration curve is illustrated in Figure 3B, which demonstrates that the predictive results of the nomogram were consistent with the actual probability of death.

### Independent risk factors associated with ICU admission

**Figure 4:**
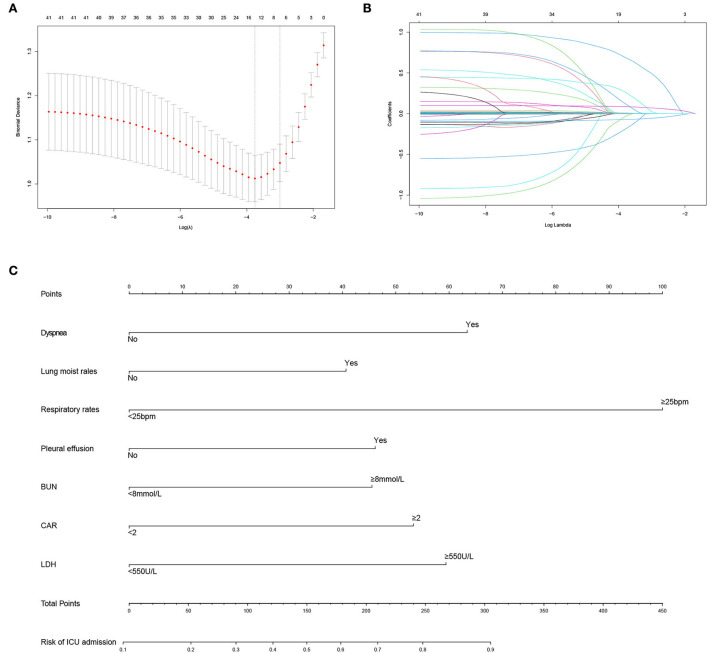
Predictor selection and prognostic nomogram development for predicting ICU admission of patients with PCP. **(A)** Identification of optimal parameters (lambda) in the LASSO model using minimum criteria and 5-fold cross-validation. Dotted vertical lines are drawn at the selected values using the minimum criteria and the 1 standard error of the minimum criteria (1-SE criteria); **(B)** LASSO coefficient profiles of 41 features; **(C)** prognostic nomogram formulated for predicting ICU admission of patients with PCP. LASSO, least absolute shrinkage and selection operator; ICU, intensive care unit; PCP, *Pneumocystis jirovecii* pneumonia; bpm, beat per minute; BUN, blood urea nitrogen; CAR, C-reactive protein/albumin ratio; LDH, lactate dehydrogenase.

The early and available factors were included in LASSO regression analysis for predicting ICU admission (Figures 4A,B). The results showed that respiratory rate, dyspnea, lung moist rales, heart rate, LDH, BUN, CAR, and pleural effusion were potential predictors (Table 5). These factors also showed significant differences between the ICU inpatients and non-ICU inpatients in the univariable analysis (Table 3). For the ease of use, the cutoff values of the variables respiratory rate, BUN, CAR, and LDH were 25 bpm, 8 mmol/L, 2 mg/g, and 550 U/L, respectively. The AUCs, specificity, and sensitivity of all the predicting factors are given in Table 5.

**Table 5:** ROC analysis and multivariable logistic regression analysis of the selected risk factors of ICU admission of patients with PCP identified by LASSO regression.

**Variables**	**ROC curve analysis**				**Multivariable logistic**		
					**regression analysis**		
	**AUC**	**Cutoff**	**Specificity**	**Sensitivity**	**β**	**Odds ratio (95%CI)**	***P* value**
Dyspnoea, yes vs. no	0.613	–	0.33	0.90	0.314	2.440 (1.324–4.548)	**0.004**
Lung moist rales, yes vs. no	0.586	–	0.54	0.63	0.631	1.879 (1.160–3.062)	**0.011**
Respiratory rates (bpm), ≥25 vs. < 25	0.744	24.5	0.81	0.59	1.325	3.763 (2.192–6.631)	**< 0.001**
Heart rates (bpm), ≥100 vs. < 100	0.612	102.5	0.75	0.46	0.404	1.498 (0.901–2.503)	0.112
Pleural effusion, yes vs. no	0.565	–	0.75	0.38	0.640	1.896 (1.141–3.199)	**0.015**
BUN (mmol/L), ≥8 vs. < 8	0.586	8.0	0.71	0.48	0.629	1.875 (1.151-3.081)	**0.012**
CAR (mg/g), ≥2 vs. < 2	0.707	1.76	0.69	0.63	0.703	2.020 (1.245–3.289)	**0.004**
LDH (U/L), ≥550 vs. < 550	0.695	545.5	0.67	0.65	0.862	2.367 (1.463–3.854)	**< 0.001**

ROC, receiver operating characteristic; ICU, intensive care unit; PCP, *Pneumocystis jirovecii* pneumonia; LASSO, least absolute shrinkage and selection operator; AUC, area under curve; CI, confidence interval; bpm, beat per minute; BUN, blood urea nitrogen; CAR, C-reactive protein/albumin ratio; LDH, lactate dehydrogenase. The bold values indicates the value of *p* < 0.05 and the difference which are considered as statistically significant.

In the multivariate logistic regression analysis, the results revealed that dyspnea (OR = 2.440, 95% CI: 1.324–4.548, *P* = 0.004), lung moist rales (OR = 1.879, 95% CI: 1.160–3.062, *P* = 0.011), respiratory rate (OR = 3.763, 95% CI: 2.192–6.631, *P* < 0.001), pleural effusion (OR = 1.896, 95% CI: 1.141–3.199, *P* = 0.015), BUN (OR = 1.875, 95% CI: 1.151–3.081, *P* = 0.012), CAR (OR = 2.020, 95% CI: 1.245–3.289, *P* = 0.004), and LDH (OR = 2.367, 95% CI: 1.463–3.854, *P* < 0.001) were independent predicting factors affecting ICU admission (Table 5), and the parameter heart rate was excluded (*P* = 0.112).

### Construction and validation of the tool for the probability of ICU admission

**Figure 5:**
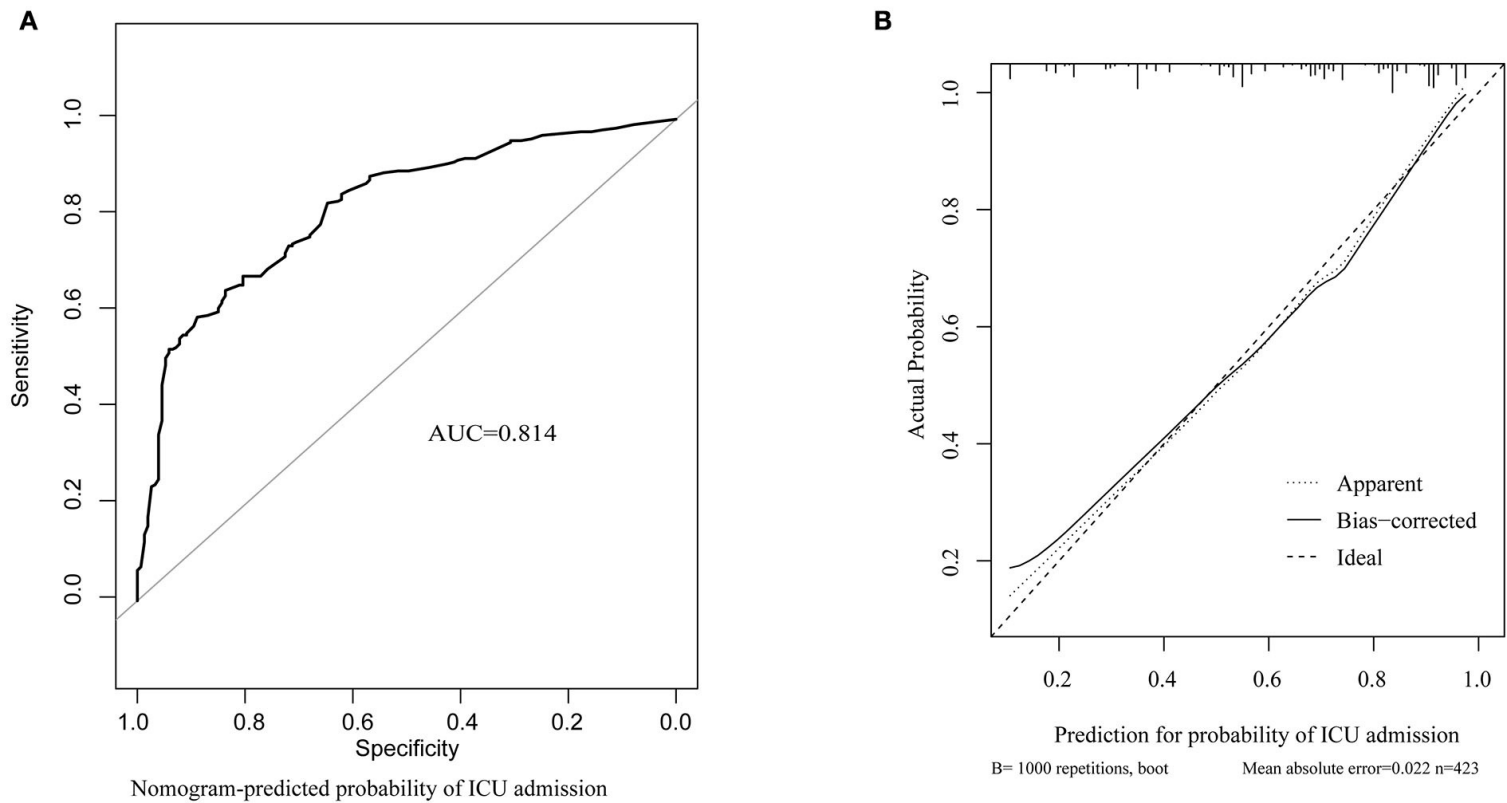
Predictive power assessment of the tool for predicting ICU admission in the validation. **(A)** ROC curve and AUC of predicting ICU admission. **(B)** calibration curves for predicting the probability of ICU admission. The dotted line represents a perfect prediction by an ideal model, and the solid line represents the performance of the nomogram in the validation. ICU, intensive care unit; ROC, receiver operating characteristic; AUC, area under curve.

The predicting tool was constructed based on the seven independent high-risk factors and is displayed as a nomogram in Figure 4C. For validation, the AUC value of this nomogram was 0.814 (95% CI: 0.773–0.856) in the ROC curve (Figure 5A), and the C-index was 0.804 for the 1,000 samples from bootstrap resampling. The good C-index and AUC value were enough to show the relatively good discrimination powers of this tool. The prognostic nomogram also performed well in predicting ICU admission accuracy, as supported by the good calibration curve (Figure 5B).

**Figure 6:**
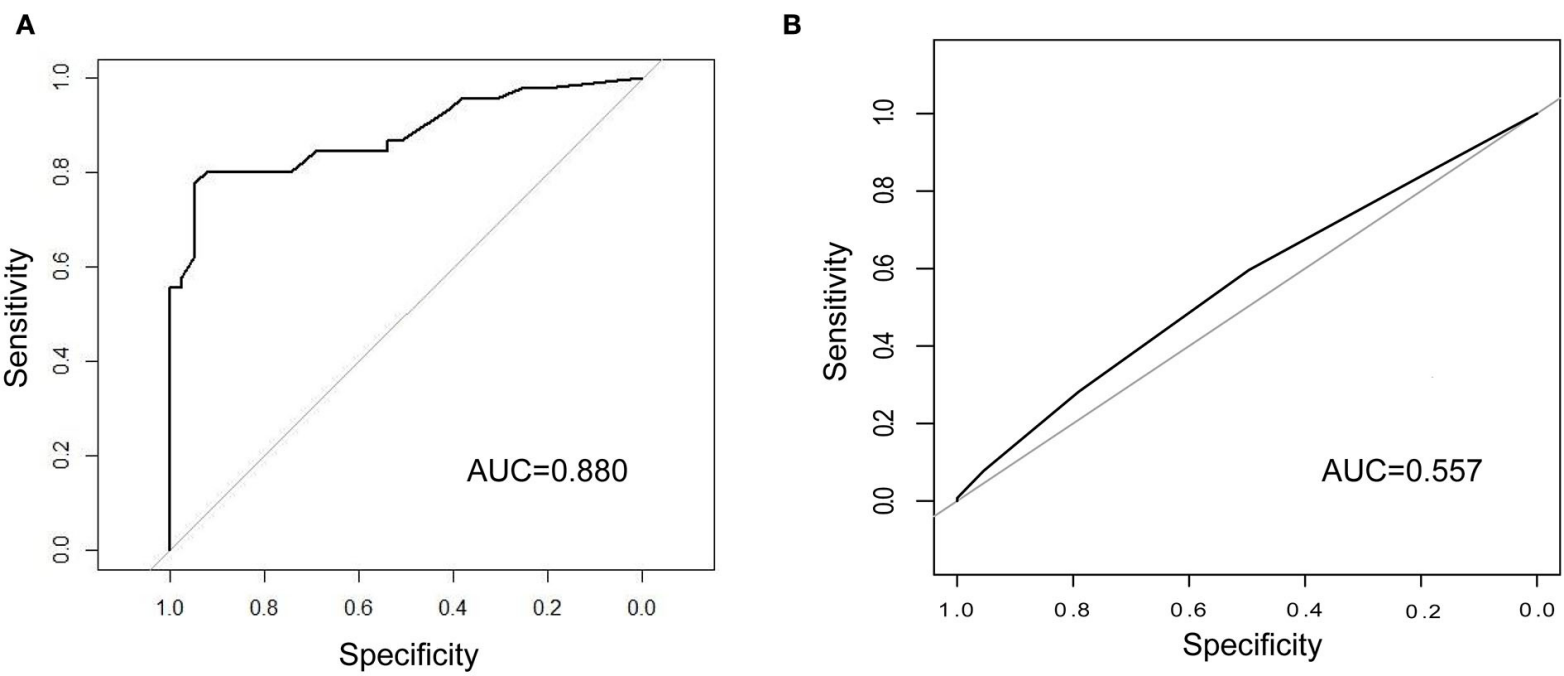
Comparison of the predictive power between our tool and CURB-65 score. **(A)** ROC curve and AUC of predicting ICU admission in the external validation; **(B)** ROC curve and AUC of CURB-65 score for predicting ICU admission in our patients. ICU, intensive care unit; ROC, receiver operating characteristic; AUC, area under curve.

In the external validation (Figure 6A), the AUC value of the nomogram was 0.880 (95% CI: 0.806–0.955). The calibration curve was unfit, which showed that the predicting accuracy may need to be improved. Then, we conducted a comparison of the predictive powers of the CURB-65 score and our tool.

### Comparison of the CURB-65 score and our tool

The CURB-65 score is a clinical tool to evaluate the demand for ICU admission of patients with CAP. Patients whose CURB scores were 3 or higher were advised to be transferred to the ICU. Of the 423 patients, over half of the patients (55.8%) were transferred to the ICU. The differences in the CURB scores between the non-ICU inpatients and ICU inpatients were not significant. Regarding predictive powers (Figures 6A,B), the AUC value of our tool for predicting ICU admission was also much higher than that of the CURB-65 score (0.880 vs. 0.557). Moreover, our tool was more sensitive than the CURB-65 score (LR-Chisq 124, 6 d.f.; *p* < 0.001).

## Discussion

Recently, the morbidity of non-HIV-infected patients with PCP increased, and their mortality of PCP was much higher (20–40% vs. 5–15%) than that of HIV-infected patients (1, 2, 36). Previous studies have developed clinical prognostic models of HIV-infected patients with PCP (27, 37). However, no clinical tool was available for predicting the death risk of non-HIV-infected patients with PCP. Therefore, in this study, we developed and validated two clinical tools for predicting death and ICU admission risks of non-HIV-infected patients with PCP based on a large sample size. The internal validation and external validation demonstrated their good discrimination and predictive accuracy, especially in the tool for predicting death risk. In addition, our tool for predicting ICU admission risk was much more informative and accurate than the CURB-65 score. For doctors, both the tools were easy to use as the tools were developed based on common clinical indicators. To the best of our knowledge, this was the first study to establish tools for predicting the death risk and ICU admission risk of non-HIV-infected patients with PCP.

### Predicting tool for death

In this study, a practical clinical tool for assessing the death risk of patients was established. Based on this tool, doctors could obtain risk warnings and then tailor an appropriate therapy program, including empiric antibiotic treatment, quick control of underlying disease, and adjustment of the dose of corticosteroids and immunosuppressants, in a timely manner to reduce the mortality of patients with PCP.

This tool was divided into two sections. The first section included five risk factors: age, respiratory rate, chronic lung diseases, BUN, and LDH. The second section consisted of three high-risk factors: CMV infection, IMV, and shock. Doctors can assess the risk factors in the first section to tailor an appropriate therapy plan. The aim was to avoid the occurrence of CMV infections, IMV, and shock. Doctors need to diagnose and treat patients with such risk factors in a timely manner and have an earlier discussion with their relatives about the patients' high risk of death.

In total, three clinical features and two laboratory findings were found in the first section. The three clinical features were age ≥45 years, a respiratory rate ≥25 bpm, and chronic lung diseases. Age and history of chronic lung diseases were relatively easy to obtain, and their scores were stable. A previous study reported that older age and chronic lung diseases were independent risk factors for poor outcomes (16). In this prognostic tool, we found that patients with chronic lung diseases showed a higher risk of death than older patients. Chronic lung disease may contribute to harmful changes in a patient's local immune components and may damage alveolar structures and even lead to a cascade of inflammatory reactions (38). Some reports indicated that nearly 33.8% of patients with chronic lung diseases were colonized by *P. jirovecii* (33). Infection with *P. jirovecii* led to severe abnormalities in surfactant functions in the infected patients (10). Together, *the* damaged alveolar structure along with the dysfunctions of surfactants was enough to cause serious respiratory symptoms and poor outcomes. An increased respiratory rate, which is an early but easily overlooked sign, was also an independent risk factor for death in our study. Clinicians should pay more attention to this easily identifiable physical sign and use this factor to evaluate patients' death risk in a timely manner.

We found two laboratory findings associated with the outcome: BUN ≥ 9 mmol/L and LDH ≥ 550 U/L. Many studies have demonstrated the value of BUN in predicting the risk of death in patients with PCP (17), which was consistent with our results. Elevated LDH levels have also been mentioned in several studies as a diagnostic value for PCP (26). However, its predictive value for poor outcomes has rarely been reported (16, 30). LDH has special biochemical functions. It is a cytoplasmic enzyme and is activated in the pathway of anaerobic metabolism. Elevations in LDH are related to pulmonary damage, which could increase the production of intracellular and extracellular lactic acid and lead to the inhibition of immune cells to perform the cytolysis function, such as cytotoxic T lymphocytes (25). Therefore, the immune system will spend a longer time on the clearance of *P. jirovecii*, which could cause lasting lung damage and even poor prognosis. In addition, LDH was frequently tested in routine laboratory examinations. The LDH level should be considered in predicting the prognosis of patients, although its power was lower than that of BUN.

The second section composed of CMV infection, IMV, and shock. Previous research showed that CMV infection failed to increase the risk of death in non-HIV-infected patients with PCP (39). Inconsistent with other results, our study demonstrated that CMV infection could be regarded as a risk factor, although its predictive power was relatively low. The clear pathogenesis of CMV infection is still unknown. CMV infection is most commonly found in individuals with profound immunosuppression. Moreover, pulmonary infection with CMV can directly damage the structure of alveoli in immunocompromised patients. Coinfection with CMV may result in a more severe pulmonary injury than *P. jirovecii* infection alone (40). Our study confirmed the correlation between CMV infection and poor prognosis. The influence of CMV infection should be noted. In addition, we found that IMV and shock were closely related to poor prognosis, both of which were reported in previous studies. In our study, IMV was endowed with the highest scores, followed by shock. IMV in patients implied that they had already experienced severe gas exchange dysfunction without remission. Moreover, under long-term IMV, the risks of pneumothorax and secondary infections would increase (28, 29).

### Tool for predicting ICU admission

Using an appropriate monitoring level could improve the outcome of patients with PCP. The existing assessment tool, CURB-65 score, was not suitable for predicting the possibility of ICU admission (AUC = 0.557). Therefore, we developed a tool for predicting ICU admission. The tool was much more informative as it added some clinical symptoms (e.g., dyspnea and lung moist rales) and laboratory data (LDH and CAR) based on the CURB-65 score. Meanwhile, compared with the complicated pneumonia severity index with 20 variables (41), our tool was concise, and all seven categorical variables in our tool are available to clinicians in the outpatient department or emergency room.

To predict whether a patient needs to be hospitalized in the ICU, the evaluation can be divided into three steps according to this tool: The first step is to perform a physical examination, mainly focusing on the symptoms of dyspnea, respiratory rate, and lung moist rales. Based on our tool, patients with PCP requiring ICU hospitalization are more likely to have dyspnea and show a respiratory rate ≥25. Lung moist rales could be a sign of pulmonary infections and exudative changes, which might result in aggravated gas exchange dysfunction (42).

The second step was to evaluate the following laboratory data: LDH ≥ 550 U/L, BUN ≥ 8 mmol/L, and CAR ≥ 2, which were considered risk factors for ICU admission in our study. CAR can reflect the inflammatory response and nutritional status of the body, but its potential clinical significance needs further study. It has been reported that CAR can adequately predict the prognosis of patients with tumors and 90-day mortality of patients with sepsis (31, 34). A previous study also discussed the value of CAR in predicting the prognosis of HIV-infected patients with PCP (27). In our study, the predictive ability was highest when the cutoff value of CAR was 2, and this cutoff value was also close to the results of previous studies on HIV-infected patients with PCP and patients with sepsis (27, 31).

The last step is to observe whether there is pleural effusion on lung CT. Although most studies did not mention the importance of pleural effusion in predicting the prognosis of PCP (16, 32, 35), we found an article that suggested pleural effusion as a risk factor in hematologic patients with PCP (23). The atypical radiographic sign of pleural effusion may suggest that these patients are complicated with the infection of other pathogens (24) or that underlying lung disease is progressing. Pleural effusion could cause physical compression of lung tissues and then worsen gas exchange (22), aggravate dyspnea, and eventually lead to respiratory failure. Referring to the aforementioned three steps, clinicians can effectively predict the possibility of ICU admission of non-HIV-infected patients with PCP and use the appropriate monitoring level.

## Limitations

In our study, several limitations were considered. First, unlike HIV-infected patients with PCP, non-HIV-infected patients with PCP were admitted to different departments of the hospital, so it was difficult to collect data for prospective validation. However, we conducted internal validation and external validation based on the existing data, and the prediction performance of our tools was ideal. Meanwhile, we planned to collect data and update our prediction tools every few years. Second, the number of patients with organ transplantation was low, which was due to the nature of our hospital. The features of the patients in different hospitals were significantly different. Our predictive tools could be more suitable for the patient group that has a lower proportion of patients with organ transplantation, as same as the feature of patient groups in many hospitals. Third, in the validation set, the number of patients was not large, and this study evaluated medical records during the COVID-19 pandemic, when ICU-related healthcare services were extremely limited. Under the background of the prevalence of COVID-19, to a certain extent, the actual probability of ICU admission would fluctuate, which was different from the prediction results of our tool. However, with the control of COVID-19 and the increase in patients with PCP in our series studies, the accuracy of the predictive tools would be higher.

## Conclusion

In conclusion, two predictive tools for evaluating the risk of death and ICU admission of non-HIV-infected patients with PCP were developed and validated. Both the tools could provide clinicians some risk warnings. Based on this information, doctors can tailor appropriate therapy programs, use an appropriate monitoring level for patients with a high risk of death, and eventually reduce the mortality of patients.

## Data availability statement

The raw data supporting the conclusions of this article will be made available by the authors, without undue reservation.

## Ethics statement

The studies involving human participants were reviewed and approved by the Institutional Review Board of Peking Union Medical College Hospital. The Ethics Committee waived the requirement of written informed consent for participation.

## Author contributions

HL and W-cC involved in the material preparation, data collection, and analysis. FJ and HL contributed to the design of this study and drafted the initial manuscript. H-lW and JX contributed to the conception of the manuscript and interpretation of the data, and provided some valuable suggestions and revisions to the draft. All authors commented on the final versions of the manuscript. All authors approved the final manuscript and were accountable for the contents of the article.

## Funding

This study was supported by the National High Level Hospital Clinical Research Funding (grant number 2022-PUMCH-A-118).

## Conflict of interest

The authors declare that the research was conducted in the absence of any commercial or financial relationships that could be construed as a potential conflict of interest.

## Publisher's note

All claims expressed in this article are solely those of the authors and do not necessarily represent those of their affiliated organizations, or those of the publisher, the editors and the reviewers. Any product that may be evaluated in this article, or claim that may be made by its manufacturer, is not guaranteed or endorsed by the publisher.
