# Supplementary figures and images for: Development and validation of tools for predicting the risk of death and ICU admission of non-HIV-infected patients with Pneumocystis jirovecii pneumonia
Source: Front Public Health. 2022 Nov 8;10:972311. doi: 10.3389/fpubh.2022.972311 (PMC9679649; doi:10.3389/fpubh.2022.972311)

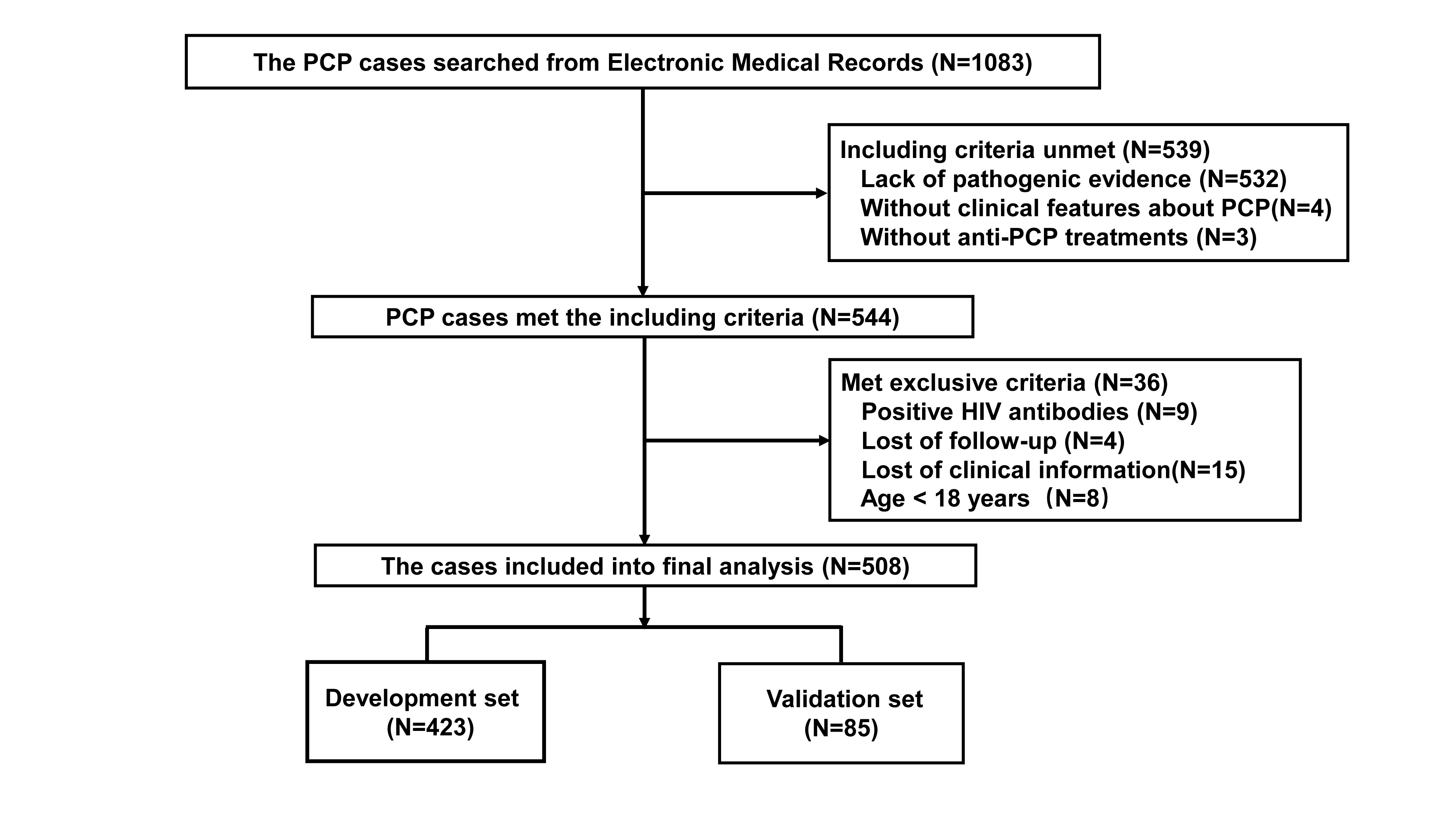

Supplement: Supplementary file 1 [file Image_1.JPEG]

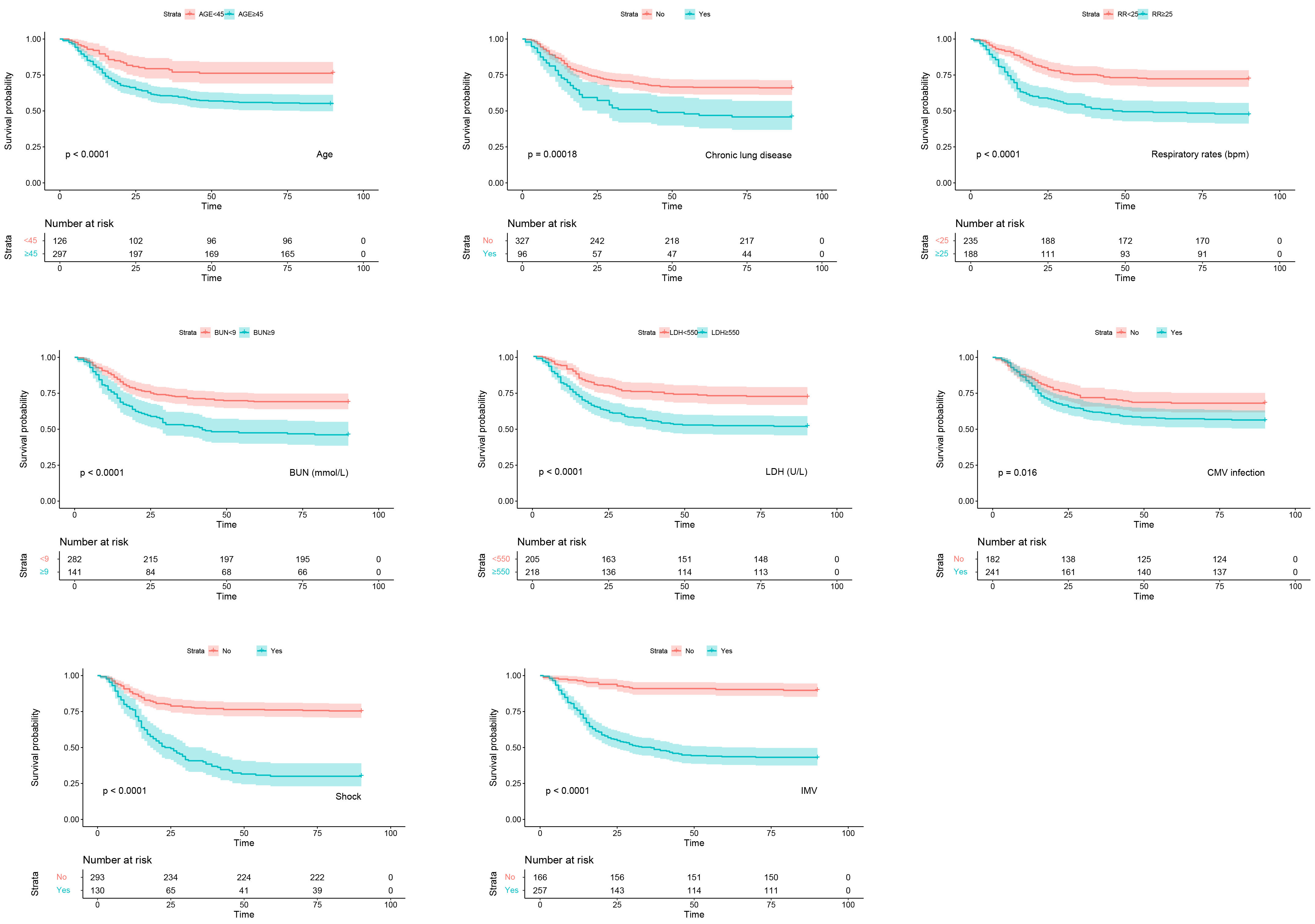

Supplement: Supplementary file 2 [file Image_2.PNG]
